# A novel splice-altering *TNC* variant (c.5247A > T, p.Gly1749Gly) in an Chinese family with autosomal dominant non-syndromic hearing loss

**DOI:** 10.1186/s12920-024-01964-x

**Published:** 2024-07-17

**Authors:** Min He, Miaomiao Hu, Qiang Zhang, Kai Yao

**Affiliations:** 1 Department of Neurology, The First People’s Hospital of Wuhu, Chizhu Shandong Road, Jiujiang District, Wuhu, 241000 Anhui Province China; 2 Key Laboratory of Digital Technology in Medical Diagnostics of Zhejiang Province, Dian Diagnostics Group Co., Ltd., Hangzhou, 310030 Zhejiang Province China

**Keywords:** Tenascin-C, Novel mutation, Non-syndromic hearing loss, Autosomal dominant

## Abstract

**Background:**

This study aims to analyze the pathogenic gene in a Chinese family with non-syndromic hearing loss and identify a novel mutation site in the *TNC* gene.

**Methods:**

A five-generation Chinese family from Anhui Province, presenting with autosomal dominant non-syndromic hearing loss, was recruited for this study. By analyzing the family history, conducting clinical examinations, and performing genetic analysis, we have thoroughly investigated potential pathogenic factors in this family. The peripheral blood samples were obtained from 20 family members, and the pathogenic genes were identified through whole exome sequencing. Subsequently, the mutation of gene locus was confirmed using Sanger sequencing. The conservation of *TNC* mutation sites was assessed using Clustal Omega software. We utilized functional prediction software including dbscSNV_AdaBoost, dbscSNV_RandomForest, NNSplice, NetGene2, and Mutation Taster to accurately predict the pathogenicity of these mutations. Furthermore, exon deletions were validated through RT-PCR analysis.

**Results:**

The family exhibited autosomal dominant, progressive, post-lingual, non-syndromic hearing loss. A novel synonymous variant (c.5247A > T, p.Gly1749Gly) in *TNC* was identified in affected members. This variant is situated at the exon–intron junction boundary towards the end of exon 18. Notably, glycine residue at position 1749 is highly conserved across various species. Bioinformatics analysis indicates that this synonymous mutation leads to the disruption of the 5' end donor splicing site in the 18th intron of the *TNC* gene. Meanwhile, verification experiments have demonstrated that this synonymous mutation disrupts the splicing process of exon 18, leading to complete exon 18 skipping and direct splicing between exons 17 and 19.

**Conclusion:**

This novel splice-altering variant (c.5247A > T, p.Gly1749Gly) in exon 18 of the *TNC* gene disrupts normal gene splicing and causes hearing loss among HBD families.

**Supplementary Information:**

The online version contains supplementary material available at 10.1186/s12920-024-01964-x.

## Background

Hearing loss significantly impacts patients' quality of life, which is mainly caused by a combination of genetic and environmental factors, with approximately 60% of cases attributed to genetics [1]. Hereditary hearing loss can be classified into syndromic hearing loss and non-syndromic hearing loss based on hearing symptoms, with non-syndromic hearing loss (NSHL) accounting for around 70% of hereditary hearing loss [2–4]. The non-syndromic hearing loss can be categorized into autosomal recessive inheritance, autosomal dominant inheritance, X-chromosome linked inheritance, and mitochondrial maternal inheritance, based on different modes of transmission. With the rapid development of molecular sequencing technology, especially whole exome sequencing (WES), more genes related to autosomal dominant non-syndromic hearing loss (ADNSHL) have been discovered. To date, more than 79 loci for ADNSHL have been mapped to chromosomal regions and 63 genes for ADNSHL have been identified (http:// hereditaryhearingloss.org).

The *TNC* gene (NM_002160.2, NP_002151.2), located on chromosome 9q31.3-q34.3 and spanning 28.54 Mb, is responsible for DFNA56 and encodes the multifunctional hexoscan protein Tenascin-C [5]. Tenascin-C is an extracellular matrix (ECM) glycoprotein found in the basement membrane and spiral layer of the cochlear bone, consisting of a tenascin assembly (TA) domain, a linear array of epidermal growth factor-like (EGFL) repeats, a series of fibronectin type III (FN-III) domains, and a globular domain at the terminal [6]. The FN-III domain is the crucial functional component of tenascin-C, playing a significant role in auditory development and repair of hearing injuries [7]. The reporting of missense and synonymous mutations has consistently increased in recent years. Numerous studies have shown that synonymous mutations can contribute to diseases through various mechanisms, such as influencing gene splicing [8], mRNA stability [9], and protein translation rate [10]. Since 2013, *TNC* has been identified as a novel pathogenic gene associated with non-syndromic hearing loss [11]. Reports of *TNC* mutations in non-syndromic hearing loss are emerging. However, there have been no reports on synonymous mutations in *TNC* causing non-syndromic hearing loss. In this study, a five-generation Chinese family (family HBD) from Anhui Province was included, which exhibited autosomal dominant non-syndromic hearing loss. Whole exome sequencing and Sanger verification identified a novel splice-altering variant in the *TNC*, c.5247A > T (p.Gly1749Gly), within family HBD.

## Methods

### Family members and clinical data

**Fig. 1:**
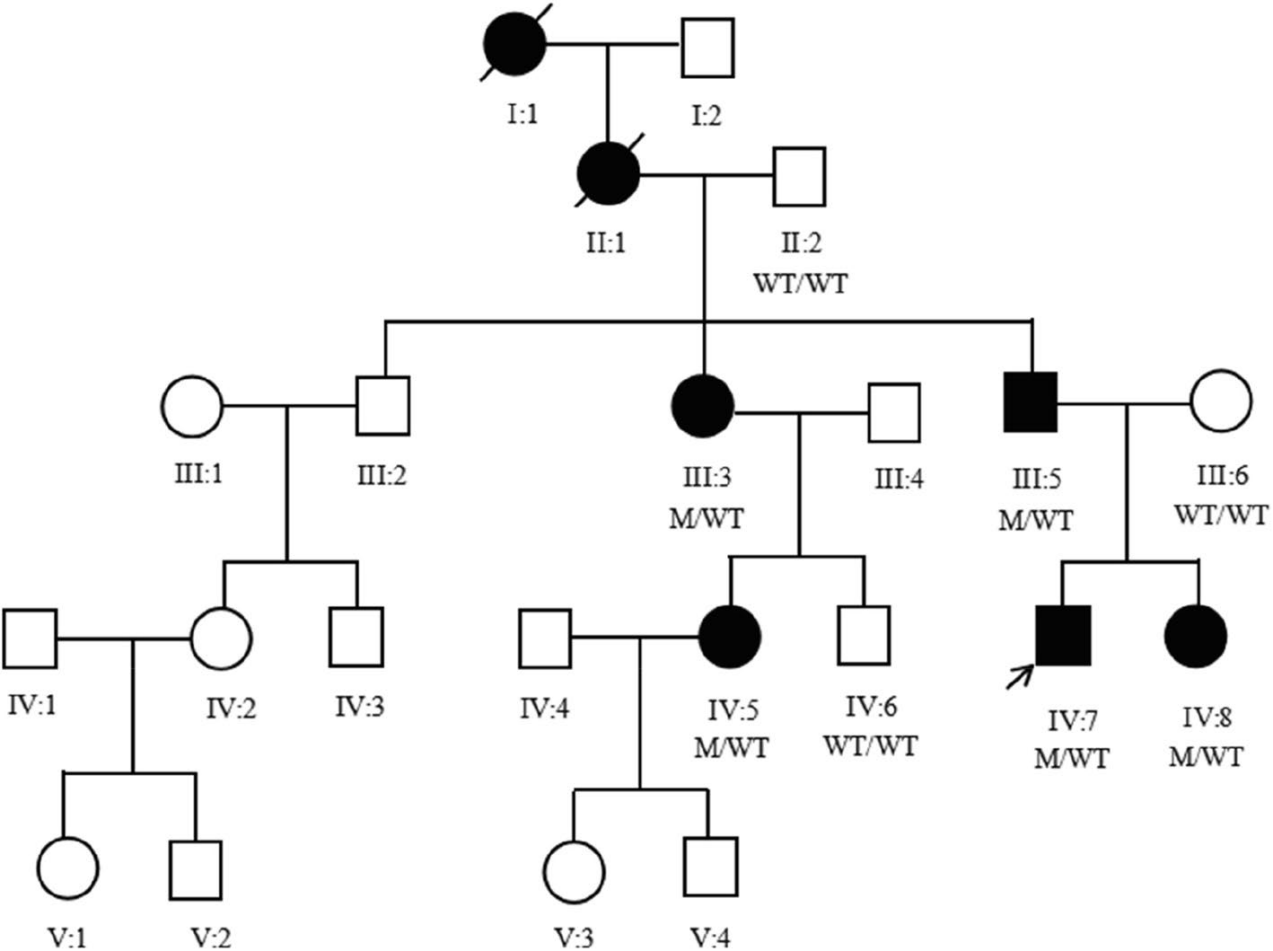
Pedigree of the Chinese family HBD. Filled symbols represent affected individuals, and empty symbols represent unaffected individuals. The square represents males and the circle represents females. The arrow indicates the proband. WT: wild-type; M: mutant

The family, referred to here as HBD, is a five-generation Chinese family from Anhui Province with autosomal dominant non-syndromic hearing loss (Fig. 1). The proband is a 23-year-old male with a family history of hearing loss. This family has autosomal dominant, progressive, post-lingual, non-syndromic sensorineural hearing loss, and these features are consistent with ADNSHL. A total of 20 family members participated in the study, including 12 males and 8 females. Among them were 5 patients with non-syndromic hearing loss, all of whom had moderate or higher hearing loss and did not use hearing aids or undergo surgery. Informed consent has been obtained from all participating members of the HBD family or their guardians and approved by the hospital Ethics Committee (No. YYLL20230039).

All participants underwent medical history collection, physical examination, intelligence assessment, hearing examination and genetic testing. The basic data included age of onset, disease progression, trauma history, noise exposure, utilization of ototoxic drugs, hearing aid usage, presence of tinnitus and vertigo. The physical examination included assessing the skin condition, hair quality, limb functionality, iris appearance, inner canthal distance measurement, outer ear structure and inner ear health. The Wechsler Adult Intelligence Scale-Revised in China (WAIS-RC) is used for intelligence assessment, including the evaluation of full intelligence quotient (FIQ), verbal intelligence quotient (VIQ), and performance intelligence quotient (PIQ).

The hearing examination includes pure tone audiometry (PTA), auditory brainstem response (ABR), acoustic immittance measurement, tinnitus detection, distortion product otoacoustic emissions (DPOAE), computed tomography (CT) scan of the temporal bone and vestibular system. In order to determine the degree of hearing loss in patients, PTA is employed to assess the average air conduction thresholds at frequencies of 250, 500, 1000, 2000, 4000 and 8000 Hz. To conduct ABR testing, the skin should be cleansed using an alcohol swab, followed by placement of two electrodes on the midline of the forehead and vertex. Subsequently, the patient should assume a supine position. A click stimulus with an intensity level of 70 dB was selected for testing at a rate of 21.1 stimuli per second. ABR testing is conducted on both ears of each participant to automatically measure amplitude and latency values for waves I, III, and V, as well as interpeak latencies for waves I-III, III-V, and I-V in order to assess potential lesions along the auditory pathway from cochlea to brainstem. DPOAE is an objective indicator of cochlear and outer hair cell functionality. Examinations were conducted using the AccuScreen 42D automatic otoacoustic emission tester (Madsen, Denmark) in a low-noise environment with background noise levels ≤ 40 dB. The tested frequency range included 1.5, 2.0, 3.0, 4.0, 5.0, and 6.0 kHz frequencies. Signal amplitude and signal-to-noise ratio were recorded at each frequency to calculate the pass rate. The severity of hearing loss was defined as mild (26–40 dB), moderate (41–55 dB), moderately severe (56–70 dB), severe (71–90 dB), or profound (> 90 dB) [11]. Furthermore, high resolution computed tomography (HRCT) was also performed on five affected individuals.

### DNA extraction

The peripheral blood samples of 20 participants from the HBD family were collected, and DNA was extracted from the peripheral blood using QIAamp DNA Blood Midi KIT (Qiagen, Dusseldorf, Germany), the purity and concentration of the extracted DNA were determined using a Nanodrop2000 ultramicrospectrophotometer.

### Screening for mutations in common hearing loss gene

The Genetic Deafness Microarray was utilized to identify common deafness genes in families III:3, III:4, III:5, III:6, IV:5, IV:6, IV:7, IV:8. These included *GJB2*, *GJB3*, mitochondrial *MT-RNR1*, and *SLC26A4* [12].

### Whole exome sequencing

The IDT xGen Exome Research Panel v1.0 kit was used to capture exon regions, including the DNA coding region and adjacent shear region of genes associated with hereditary hearing loss. Dian Diagnostics Group Co., Ltd., completed the sequencing process using the NovaSeq 6000 platform (Illumina, San Diego, CA, USA).

The initial sequencing data obtained were processed according to the following criteria: 1) filtering out sequences with low quality; 2) removing sequencing adapters and primer sequences; 3) excluding regions with poor base quality to eliminate any chaotic sequences. This was followed by aligning against the GRCh37/hg19 reference sequence using Burrow-Wheeler Aligner (BWA) software [13]. Subsequently, duplicate sequences were removed using the Picard tool [14]. Next, Genome Analysis ToolKit (GATK) software is employed for mutation site detection, followed by annotation of these sites using snpEFF software [15]. The database includes 1000 Genome, NHLBI Exome Sequencing Project, ClinVar, and dbNSFP. The prediction tool for identifying harmful genetic mutations includes MutationTaster [16], dbscSNV_AdaBoost [17], dbscSNV_RandomForest [18], NNSplice [19], and NetGene2 [20].

### Sanger sequencing

Following whole exome sequencing, candidate genes were isolated and subjected to Sanger sequencing analysis. Through PCR amplification and sequencing, the mutation can be detected and identified. The primers for *TNC* gene mutation sites have been designed and synthesized as follows: 5'-ATTCCTTGGAGTCAGCTA-3' and 5'-CCAGTAGGTCTTGTTCAA-3'. Subsequently, these primers were synthesized by Sangon Biotech (Shanghai) Co., Ltd.

### Evolutionary conservation analysis

To determine the phylogenetic conservation of the variant, Clustal Omega software were used to align the sequence of Homo sapiens (human) with a range of different mammals such as Macaca fascicularis (monkey), Pan troglodytes (chimpanzee), Halichoerus grypus (seal), Marmota monax (marmot), Ursus arctos (bear), and Delphinus delphis (delphinus) [21].

### Splicing site mutation validation

In order to investigate the impact of the identified variant (c.5247A > T, p.Gly1749Gly) on splice sites, we performed RT-PCR amplification of exons 17–20 of the *TNC* gene and subsequently using agarose gel electrophoresis. RNA extraction was performed on fresh blood samples obtained from individuals III:3, III:4, III:5, III:6, IV:5, IV:6, IV:7, and IV:8 utilizing a spin column-based blood total RNA purification kit (Sangon Biotech, Shanghai, PR China). Following this step, the obtained total RNA underwent reverse transcription using the iScript Reverse Transcription Supermix for RT-qPCR Kit (Bio-Rad, USA). Using cDNA as a template, the exons 17–20 sequences were amplified using the following primer sequences: forward 5'-AAGCCGAACCGGAAGTTGACAACC-3' and reverse 5'-CTTTCTCGCCTGTGTAGGAGATGA-3'. Subsequently, electrophoresis was conducted on a 2% agarose gel at 95 V for 30 min, and the results were visualized under UV illumination.

The real-time fluorescence quantitative PCR method was employed to quantify the expression levels of bands amplified, with all reactions being performed in triplicate.

## Results

### Clinical features

The HBD family, which consisted of 7 patients with hearing loss, including 2 deceased individuals (I:1, II:1), ultimately comprised 5 affected and 15 unaffected individuals. The age at onset of hearing impairment ranged from 6 to 16 years. The hearing examination and clinical data of the affected members in HBD showed progressive, post-lingual, non-syndromic sensorineural hearing loss. Notably, there is no history of trauma, noise exposure, or ototoxic drug use. The Affected members showed no abnormalities during the physical examination and intelligence assessment. However, slight speech expression abnormalities were observed in the proband (IV:7) and their sister (IV:8) (Table 1).

**Table 1:** Clinical characteristics of patients with *TNC* variants in this study

Family members	III:3	III:5	IV:5	IV:7	IV:8
Sex	Female	Male	Female	Male	Female
Age	52	48	28	23	15
Age of onset (years)	16	10	12	6	6
Severity of hearing loss	Severe	Severe	Moderately severe	Moderately severe	Moderately severe
Verbal expression	Normal	Normal	Normal	Minor anomaly	Minor anomaly
Intelligence assessment	Normal	Normal	Normal	Normal	Normal
ABR	Normal	Normal	Normal	Normal	Normal
DPOAE	Absent at all frequencies	Absent at all frequencies	Absent at all frequencies	Absent at all frequencies	Absent at all frequencies
General physical examination	Normal	Normal	Normal	Normal	Normal
Vertigo (Yes or No)	No	No	No	No	No
Tinnitus (Yes or No)	No	No	No	No	No
Exposure to noise (No or Yes)	No	No	No	No	No
Ototoxic drugs expose (No or Yes)	No	No	No	No	No

**Fig. 2:**
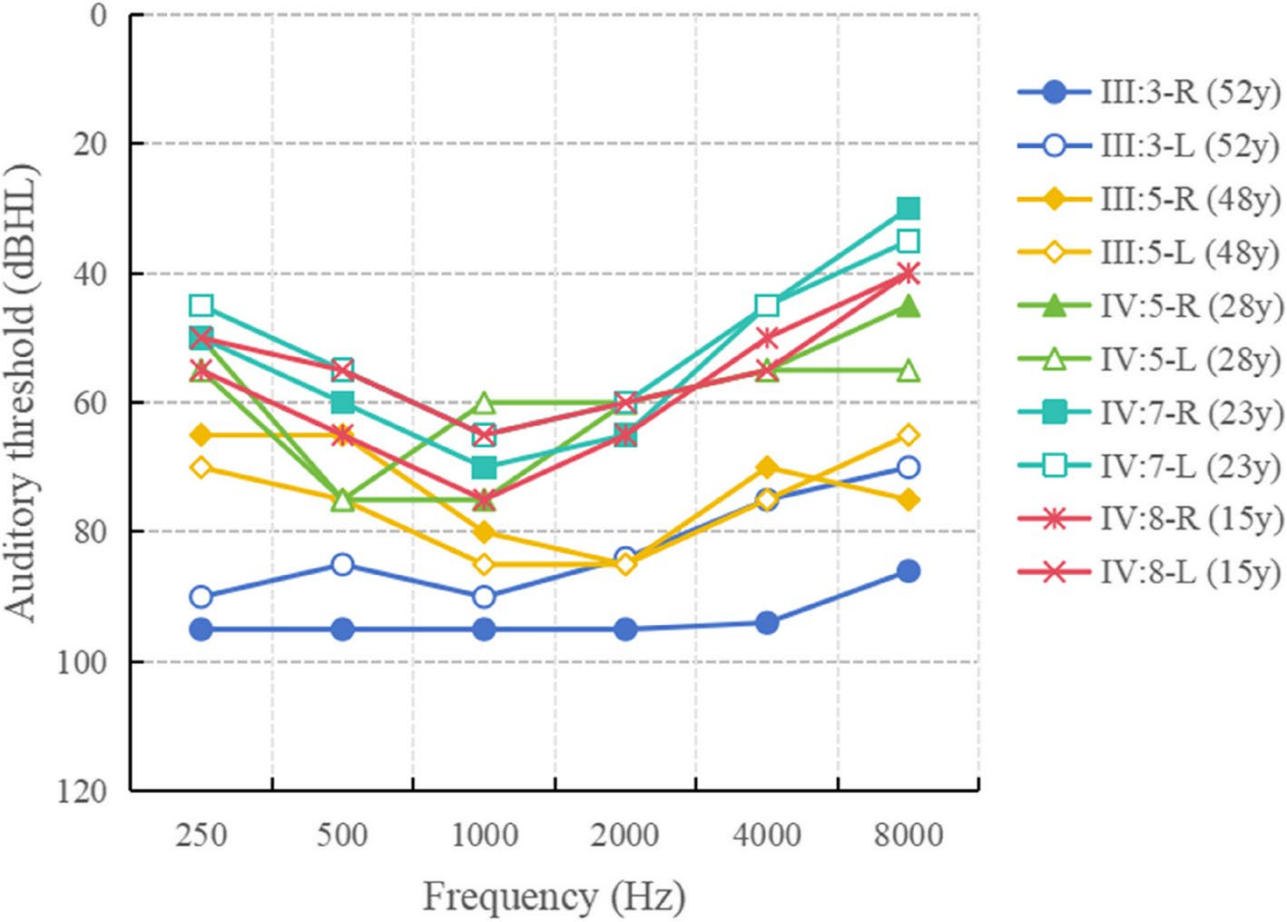
Pure-tone audiograms of the affected members in HBD. dB, decibels; Hz, Hertz; y, years

The affected individuals initially had low frequency hearing loss, which gradually worsened as they aged (Fig. 2). No vertigo or tinnitus was reported in affected members of HBD. The ABR test results were normal for the affected members, but DPOAE testing revealed cochlear dysfunction. High resolution CT scans showed normal structure in the middle and inner ear (Table 1).

### WES identifies a causative variant in TNC

**Fig. 3:**
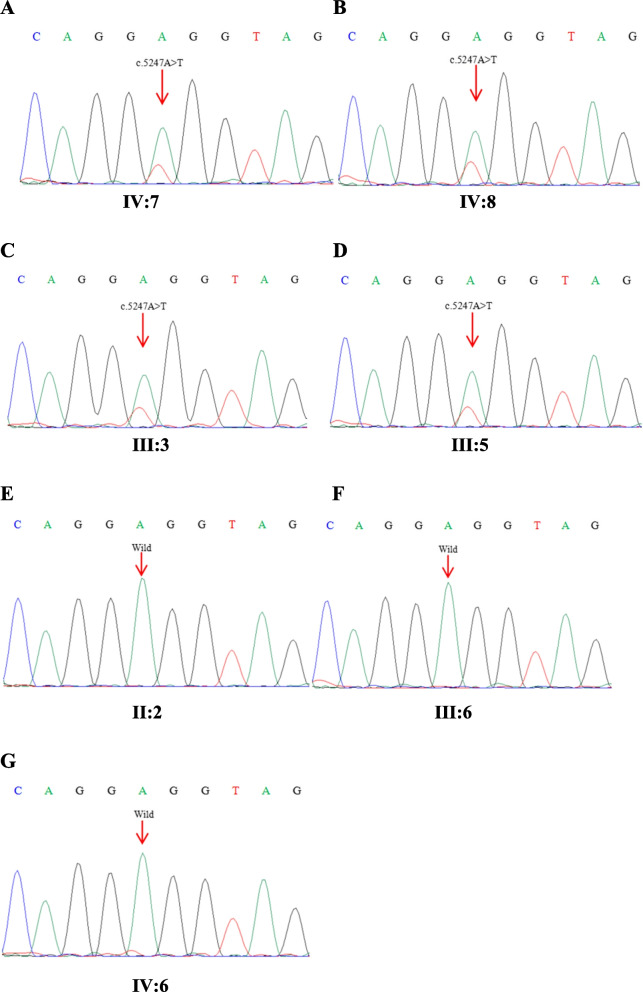
The sanger sequence results of the variant c.5247A > T in HBD family. Arrows indicate the position of the nucleotide changes identified

To identify the genetic cause of hearing loss, a common genetic test for deafness is conducted utilizing a genetic deafness chip. The findings do not reveal any variants in *GJB2*, *GJB3*, *SLC26A4*, and mitochondrial *MT-RNR1*. Consequently, WES was performed on four affected individuals (III:3, III:5, IV:7, IV:8) and three unaffected individuals (II:2, III:6, IV:6) in HBD. WES analysis successfully identifies a novel synonymous variant (c.5247A > T, p.Gly1749Gly) located in exon 18 of the *TNC* gene among the four affected family members. Subsequent Sanger sequencing confirms that this variant in *TNC* (c.5247A > T) co-segregates in the family (Fig. 3). This variant was detected in individuals IV:7, IV:8, III:3, and III:5, all of whom were diagnosed with hearing loss (Fig. 3A-D). The variant was not detected in individuals II:2, III:6, and IV:6, who exhibited normal hearing abilities (Fig. 3E-G). Furthermore, this variant was not present in the 1000 Genomes database, NHLBI Exome Sequencing Project, and ClinVar. In addition to the *TNC* gene mutation, WES testing of the proband revealed mutations in genes *MYO3A* (c.610G > A, c.4484G > A), *CDH23* (c.574G > C), and *DMXL2* (c.364 + 8G > A). However, these mutations did not correspond with the family members' inherited deafness, and none of the other affected family members had these mutations, so we decided to exclude them.

### Pathogenicity and evolutionary conservation analysis

**Fig. 4:**
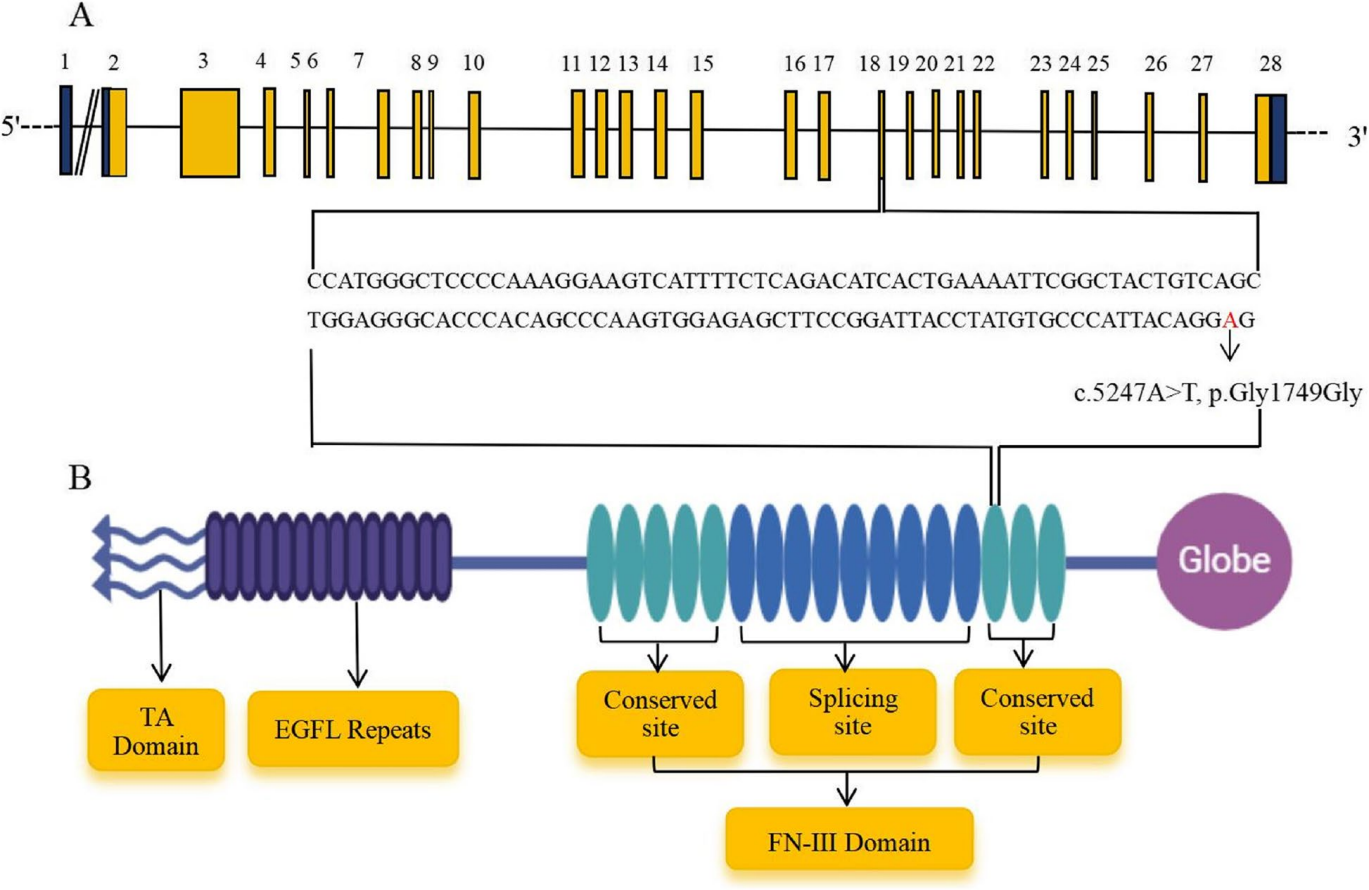
Gene mapping of HBD family with non-syndromic hearing loss. **A** Structure of *TNC* gene. **B** Structure of *TNC* translated protein*.* Mutations of c.5247A > T (p.Gly1749Gly) identified in *TNC* gene is located in exon 18, which corresponds to the13th FN-III domain in the translated protein

**Fig. 5:**
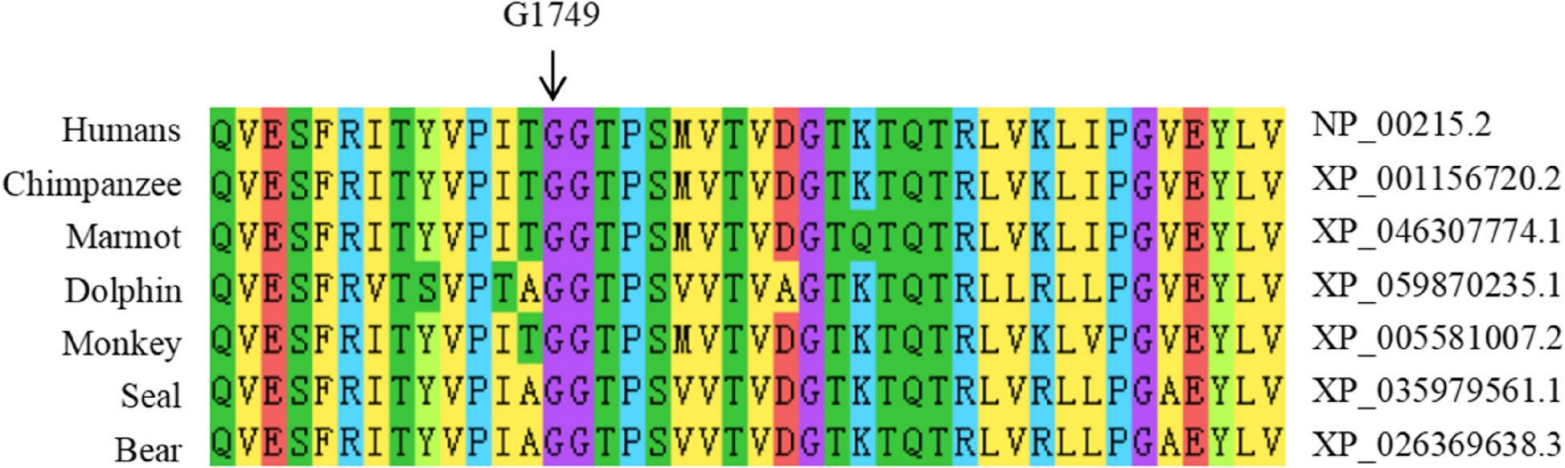
Evolutionary conservation of the Gly1749 residue of TNC across a range of species. Protein alignment shows high conservation for the position 1749 in the *TNC* protein among different species

The pathogenicity analysis for this variant was determined based on various tools, including dbscSNV (likely pathogenic) and Mutation Taster (disease causing) in Table 2. The impact of this variant on splicing was assessed using various computational tools, including NNSplice and NetGene2. Predictions from these tools suggest that this synonymous variant leads to the disruption of the 5' end donor splicing site in the 18th intron of the *TNC* gene (Table 2). This variant is located at the nucleotide end of exon 18, which corresponds to the boundary of the exon–intron junction point, specifically within the 13th FN-III domain in the translated protein (Fig. 4). Mutations occurring at this site have the potential to disrupt normal splicing and alter downstream splicing events (Table 2). Notably, glycine residue at position 1749 is highly conserved across a range of different species (such as monkey, chimpanzee, seal, marmot, bear, and dolphin), as depicted in Fig. 5.

**Table 2:** In silico prediction of the *TNC* synonymous variant c.5247A > T

Tools	Outcome	Score	Interpretation
Mutation Taster	Disease causing	1	Alteration within used splice site, likely to disturb normal splicing
dbscSNV_AdaBoost	Likely pathogenic	0.999	Splice-altering
dbscSNV_Random Forest	Likely pathogenic	0.988	Splice-altering
NNSplice	/	0.85	Donor splicing site is missing
NetGene2	/	0.88	Donor splicing site is missing

### Splicing site mutation analysis

**Fig. 6:**
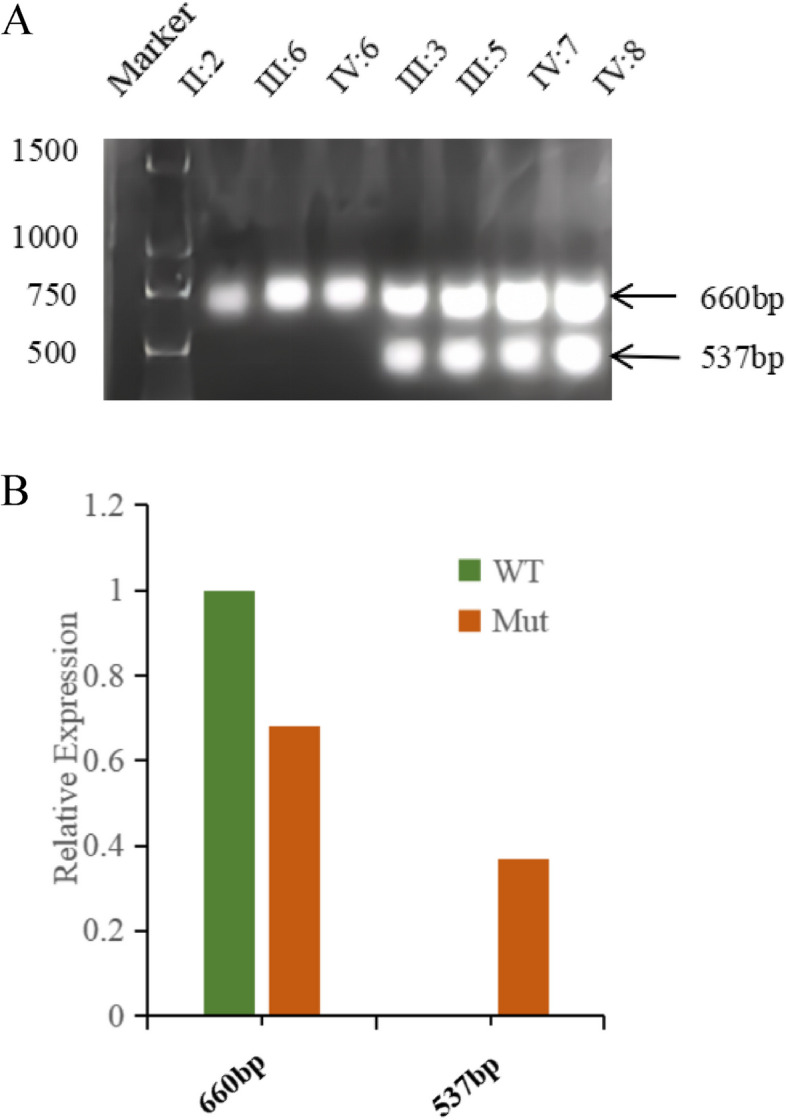
Splicing Site Mutation Analysis for c.5247A > T in *TNC*. **A** Agarose gel showing the result of the RT-PCR. **B** Relative expression of bands

In order to validate the impact of this splice-altering variant, we performed RT-PCR amplification of exons 17–20 sequences to confirm aberrantly spliced RNA presence in affected members. Agarose electrophoresis revealed that the four affected members showed two distinct bands on RT-PCR, 660 bp and 537 bp respectively, whereas normal individuals displayed only a single band (Fig. 6A). The results demonstrate that this splice-altering variant affects the splicing process of the *TNC* gene, causing exon skipping in exon 18. Furthermore, this observation was further supported by quantifying expression levels across different bands (Fig. 6B).

## Discussion

In this study, a novel splice-altering variant (c.5247A > T, p.Gly1749Gly) in the *TNC* was identified in a Chinese family with non-syndromic hearing loss. The glycine at position 1749 is conserved across a range of different species. Although this mutation does not result in an amino acid change, it has the potential to disrupt normal splicing and alter downstream splicing events, potentially impacting the function or structure of the gene.

Tenascin-C is a member of extracellular matrix glycoprotein, which exists in the basement membrane and spiral layer of cochlear bone, and is one of the main components of the basement membrane of mammals [22]. The *TNC* plays a role in regulating fluid and ion transport between endolymphatic and exolymph. Any mutation in the *TNC* may disrupt basement membrane ion homeostasis [23]. The hearing loss resulting from *TNC* mutation is classified as low-frequency non-syndromic hearing loss. Below 1000 Hz, the hearing loss is severe, gradually decreasing and returning to normal as the frequency increases. Therefore, it is crucial for patients to undergo effective early diagnosis before the progression of hearing loss to severe hearing loss [24, 25].

Since the discovery in 2013 that *TNC* mutation is implicated in non-syndromic hearing loss [26], *TNC* has been included in the non-syndromic hearing loss gene database. However, due to the rarity of *TNC* mutation, there remains a scarcity of reports on non-syndromic hearing loss attributed to *TNC* mutation. In 2013, Zhang et al. initially identified through genetic linkage analysis that two missense mutations (c.5317G > A, p.V1773M and c.5368A > T, p.T1796S) in the *TNC* gene were associated with autosomal dominant hearing loss, thereby confirming *TNC* as a novel gene implicated in hearing loss [26]. The report indicates that the *TNC* mutations (c.5317G > A, p.V1773M and c.5368A > T, p.T1796S) at these two loci are both located in exon 19, corresponding to the 13th FN-III domain. These two mutations occur at the connecting edges of two conserved domains within the FN-III structure, thereby inducing conformational changes in this region and affecting the molecular binding ability of tenascin-C. Furthermore, a case report analysis conducted in Portugal demonstrated that a 20-year-old female patient exhibited progressive bilateral hearing loss, which was ultimately attributed to a missense mutation (c.1337G > A, p.R446Q) in the *TNC* gene [24].

The disease was observed in a total of 7 individuals within this family. Following thorough hearing examinations and medical history investigations, no abnormalities were detected in skin and hair pigmentation, iris heterochromia, inner canthal distance, upper limb structure or megacolon presence. Based on these findings, it was determined that this family was non-syndromic hearing loss. None of the patients in this family had a history of trauma, noise exposure, or ototoxic drug use. The patients exhibit a genetic predisposition, with a male-to-female ratio of 2:5. Both genders are susceptible to developing the disease. The inheritance pattern followed Mendelian autosomal dominant characteristics. Therefore, this family is diagnosed with autosomal dominant non-syndromic hearing loss. In this study, we conducted a screening of four common deafness genes (*GJB2*, *GJB3*, mitochondrial *MT-RNR1*, and *SLC26A4*) in the Chinese population to exclude mutations in these commonly associated deafness gene loci. Subsequently, through whole exome sequencing, we identified a novel mutation in the *TNC* which was further confirmed by Sanger sequencing. Notably, this mutation co-segregated with the observed phenotype of familial hearing loss. To establish controls for comparison, three individuals with normal hearing from HBD family were selected and no mutations were detected at *TNC*. Therefore, it can be concluded that the *TNC* mutation is responsible for causing hearing loss in HBD families.

The N-terminal EGF domain of tenascin-C is capable of binding and activating the EGFR located on the surface of muscle stem cells, thereby facilitating the proliferation of muscle stem cells [27]. The current evidence demonstrates that tenascin-C has the ability to both promote and inhibit the growth of various neurons and neurites [28]. Considering the primary role of the tenascin-C in the human body, it is hypothesized that tenascin-C primarily functions in repairing the basement membrane in the cochlea. Following a mutation in *TNC*, the repair mechanism for inner ear damage becomes impaired, leading to a gradual accumulation of hearing impairment [26]. Therefore, the hearing impairment in affected individuals within this family is initially characterized by low-frequency hearing loss, which progressively worsens with age.

The amino acid sequence remains unchanged by synonymous mutations, and it was initially believed that a substantial number of these mutations lacked biological significance. However, numerous studies have demonstrated that synonymous mutations can induce disease by impacting precursor mRNA splicing, mRNA structural integrity and stability, as well as protein translation efficiency and speed. The most prevalent mechanism involves interference with precursor mRNA splicing through modulation of splicing enhancer or silencer elements, or the generation of an occult donor/receptor site, resulting in aberrant protein encoding or production of unstable abnormal mRNA [29]. Nucleotide changes near the splicing site are widely believed to cause protein dysfunction [30]. In this study, we have identified a mutation site located at the terminal nucleotide position of exon 18, precisely at the boundary of the exon–intron junction point. Mutations occurring at this specific site can significantly disrupt RNA splicing. Species conservation analysis revealed that this site was highly conserved in various species. When combined with the mutation predictions from dbscSNV, Mutation Taster, NNSplice, and NetGene2, it is speculated that the mutation occurring at this site may disrupt normal splicing and alter downstream splicing events, thereby potentially impacting protein function. Concurrently, the experimental findings demonstrated that the mutation impeded exon 18 splicing, resulting in complete exon 18 skipping and direct splicing of exons 17 and 19. Currently, the mutation site has not yet been identified in the 1000 Genomes database, NHLBI Exome Sequencing Project, and ClinVar databases. Additionally, no reports have been made regarding synonymous mutations in the *TNC* leading to non-syndromic hearing loss. The identification of this mutation would be beneficial for diagnosing *TNC*-related hearing loss.

The study still has limitations as we did not perform cDNA sequencing of exons 17–19, thus unable to determine the specific changes in protein levels. Additionally, we are unsure about the exact impact of this splice-altering variant on protein structure and function. Therefore, in future research, we will focus on addressing these shortcomings.

## Conclusions

In conclusion, our study has identified a novel splice-altering mutation (c.5247A > T, p.Gly1749Gly) in the *TNC* that is responsible for autosomal dominant non-syndromic hearing loss in HBD families. This represents the first reported instance of a pathogenic splice-altering mutation in the *TNC* within a non-syndromic hearing loss population, thereby broadening the range of pathogenic loci associated with the genetic database for non-syndromic hearing loss.

### Supplementary Information

Supplementary Material 1.

## Data Availability

Sequence data that support the findings of this study have been deposited in the NCBI ClinVar repository with the primary accession code VCV002775398.1.

## Abbreviations

NSHL: Non-syndromic hearing loss
ADNSHL: Autosomal dominant non-syndromic hearing loss
ECM: Extracellular matrix
EGFL: Epidermal growth factor-like
FN-III: Fibronectin type III
PTA: Pure tone audiometry
ABR: Auditory brainstem response
DPOAE: Distortion product otoacoustic emissions
CT: Computed tomography
HRCT: High resolution computed tomography
BWA: Burrow-Wheeler Aligner
GATK: Genome Analysis ToolKit
WES: Whole exome sequencing
PCR: Polymerase chain reaction
TA: Tenascin assembly
WAIS-RC: Wechsler Adult Intelligence Scale-Revised in China
FIQ: Full intelligence quotient
VIQ: Verbal intelligence quotient
PIQ: Performance intelligence quotient
RT-qPCR: Real-time quantitative polymerase chain reaction
