# Supplementary figures and images for: A novel splice-altering TNC variant (c.5247A > T, p.Gly1749Gly) in an Chinese family with autosomal dominant non-syndromic hearing loss
Source: BMC Med Genomics. 2024 Jul 17;17:189. doi: 10.1186/s12920-024-01964-x (PMC11256465; doi:10.1186/s12920-024-01964-x)

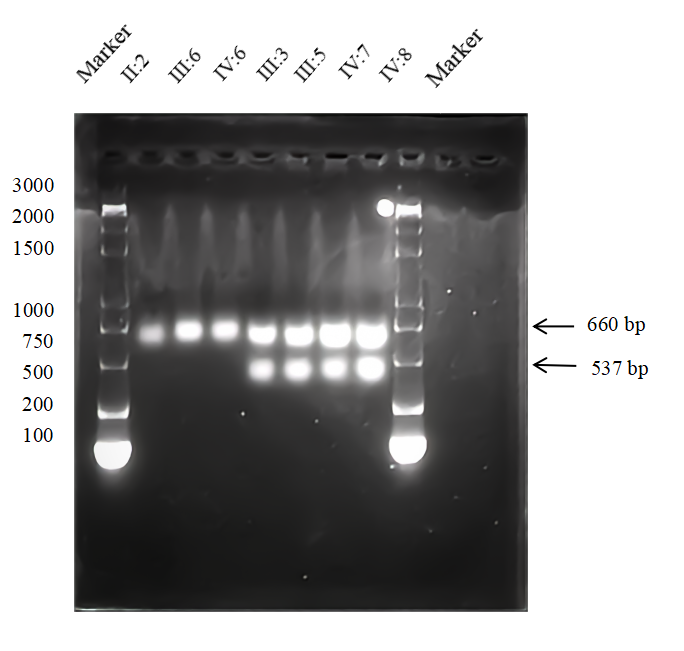

Supplement: Supplementary file 1 — Supplementary Material 1. [file 12920_2024_1964_MOESM1_ESM.doc]
